# An Analysis of the Career Pathway of Clinical Laboratory Scientists: Identifying Access Barriers and Best Practice to Increase Diversity in the Workforce

**DOI:** 10.3389/bjbs.2026.15810

**Published:** 2026-02-19

**Authors:** James A. O’Connor

**Affiliations:** Natural Sciences, School of Science, Psychology, Arts and humanities, Computer science, Engineering and Sports Science (SPACES), Canterbury Christ Church University, Canterbury, United Kingdom

**Keywords:** biomedical science, biomedical science education, biomedical science training, widening participation in biomedical science, widening participation in university

## Abstract

Widening participation among the clinical laboratory scientific workforce is essential to meet future needs of healthcare systems. The advantages of more diversity in this clinically pivotal workforce include better decision making, improved diagnostics and a wider pool of appropriately trained applicants for new and advanced posts. This review summarises a sustainable “trickle up approach” to increase diversity and widen participation at all levels of the career pathway for clinical laboratory scientists with a focus on socioeconomically disadvantaged and minoritised scientists. Issues of access to appropriate degrees are present years in advance of university application and can be addressed through meaningful outreach programmes from universities and professional bodies. Interventions to broaden degree entry access including foundation routes have proven efficacy, whereas the role of degree apprenticeships in widening participation appears to be minimal, currently. There is a higher proportion of ethnic minorities, particularly black students, who don’t complete their degree programme or attain lower awards than colleagues. Contributory factors include curriculum design along with psychosocial deficiencies in delivery. Decolonising and making biomedical science curricula and delivery more inclusive have proven effective in reducing these risks. Furthermore, socioeconomically disadvantaged students face a new challenge from generative artificial intelligence tools, where those that can pay get access to more powerful tools, creating a new gap, unless these tools are used judiciously and free at point of use. A graduate is required to complete training in a clinical laboratory to gain HCPC or equivalent registration, these places are competitive, and often unpaid. This appears to be a key barrier to widening participation, with a majority of graduates not pursuing careers as Biomedical Scientists. A state and financially supported training programme is required to broaden involvement at and post-registration. There is a paucity of information regarding the makeup of the workforce at promotional grades. However, an analysis of postgraduate study and research avenues reveals challenges for those from minoritised backgrounds and working mothers. These can be addressed through diversity in academic institutions and tailored, personalised approaches to research for working mothers to maximise participation at management and clinical leadership roles in the diagnostic laboratory.

## Introduction

There has been a concerted effort to both measure and broaden participation in healthcare professions, including the clinical laboratory, necessitated primarily to ensure adherence to equality legislation, including the UK Equality Act, 2010 [1]. The Health and Care Professions Council (HCPC), the regulator of health and care professions in the UK, first commissioned a research evaluation of Equality, Diversity, and Inclusion (EDI) information for professions under its remit in 2020 and provided an update in 2023/24 [2–4]. The data shows that the gap is gradually closing on many of the metrics that are being measured, including greater diversity and proportion of ethnicities in our clinical laboratories, more diverse age ranges and more Biomedical Scientists reporting disabilities [3, 4]. These are all positives and are following a favourable trend. However, more needs to be done particularly in areas that require further diversification and to address data gaps, particularly surrounding socioeconomic demographic makeup at point of entry to these professions [1]. There is a definite need to increase the number of Biomedical and Clinical Scientists to expertly oversee laboratory medicine departments, widening participation is a key factor in raising overall numbers of scientists while also increasing productivity [5]. In the United States, it is projected there will be a need for 5% more clinical laboratory scientists in the next 7 years [6], this situation is likely to be mirrored or amplified in many other countries including the UK and Europe. The need for more clinical laboratory scientists is punctuated by the pivotal role they play in the patient journey; actively contributing in approximately 70% of diagnoses in a healthcare system which is caring for an ageing patient population with correlated increased clinical complexity and long-term morbidity [7]. These population and clinical factors are factors which may increase the required number of future scientists, despite the modernisation of the clinical laboratory in terms of technology and automation. The variety of roles of available to clinical laboratory scientists has significantly diversified in recent years with technical, management and clinical routes to advancement now available [8], providing that prospective candidates have the necessary prerequisite knowledge and experience, which is essential to ensure optimal patient care. Against this background, it is imperative that there is a continued push to improve and widen participation in this clinically critical profession. A more diverse clinical scientific workforce has several benefits including improved diagnostics, communications and decision making [9, 10]. This review will analyse key points in the career pathway of Biomedical and Clinical Scientists, detail current trends, identify the current barriers and solutions to overcome these, including undergraduate access, attrition and attainment, registration and training routes, and postgraduate opportunities. The primary focus will be on increasing diversity in terms of among ethnic minorities and socioeconomic disadvantaged cohorts but will examine barriers that other minorities may face gaining access to the clinical laboratory scientific workforce.

## Undergraduate Biomedical Science Degree Access- Cracks in the Foundation?

The first step in laying the foundations for as wide participation as possible in Biomedical Sciences is, arguably, entry to an appropriate degree programme. There are several barriers that hinder access to an undergraduate degree, the most significant barrier being socioeconomic background [11, 12]. Analysis of attainment at secondary school using the General Certificate of Secondary Education (GCSE) is required to understand how socioeconomic background has become such a significant barrier to access to university. The Educational Policy Institute, London, publishes annual figures highlighting the attainment gaps at various educational intervals for England. Their most recent report for 2025 showed a slight decrease in the disadvantage gap at GCSE English and Maths for 2024 but is still very high at 19.1 months [13]. This gap is further highlighted when examining the progression to higher education (HE) for students from financially disadvantaged backgrounds in England. The most recent figures show a lower rate of progression across all ethnic minorities who are eligible for free school meals (FSM) (i.e., those in receipt of state welfare or with very low incomes) versus those that are not eligible for FSM [14], this is just one example of where intersectional disadvantage results in a negative compound effect.

It has been shown that students whose backgrounds are both of minority ethnicities and economic disadvantage are less likely to do well in science subjects than their peers. The English 2024/25 figures show that for students categorised as “Disadvantaged” and of either Bangladeshi, Pakistani or Black (African/Caribbean/Black British) ethnicities had the highest rates of students not achieving the expected standards at 17%, 22.9% and 22.5% respectively [15]. Furthermore, these student cohorts are at highest risk of longstanding extreme poverty regardless of educational attainment due to factors such as employment patterns (under-employment and contract types) and housing (high tenancy rates and geography) [16]. A 2021 Austrian intersectional study examined the factors that motivate students, of primarily school going age, to connect and interact with science as a broad academic discipline. Their results showed that a student’s environment plays a significant role on their enjoyment of learning science with school experience, attitudes of family and friends being the most significant contributors on how students connect with science [17]. These motivating factors can be deficit or absent for students who experience poverty and are also minoritised in other ways including ethnicity and disability, through no fault of their own. Students from poor areas are more likely to have a less meaningful and immersive experience with science at school than those from more affluent areas, it has been shown that schools within the poorest local authorities in England have experienced larger and more disproportionate cuts (due to higher debt burden and lower opportunity to raise funds from parents) in spending than schools in other local authorities [18]. When budgets are reduced, either through austerity or indirectly as seen currently, as a result of inflation, there are difficult decisions to be made by school leaders which may include reduced provision of relatively high-cost areas like science education. If a student can’t experience and interact with science in a meaningful way, they are far less likely to be motivated to continue to study science at A-level or equivalent and are thus far less likely to choose science at university [19]. Universities can play a valuable role in this regard by bridging the gap and offering schools who do not have laboratory space the opportunity to utilise laboratory space to improve student’s experience of science and to act as a motivator for students to pursue science at higher education. In a US setting, this schoolwide approach has shown that students in schools that participated performed better than students from similar schools with targeted outreach programmes, and these students were six-percent more likely to continue into higher education than students in schools that have no formal outreach programmes [20]. This disadvantage attainment gap is not a recent development nor unique to England, Wales [21] or Northern Ireland [22]; it is mirrored in many other jurisdictions and has been commonplace since the massification of Western higher education in the late 20th century [23]. This accentuates the need for targeted action before university entry as an initial step in widening access and participation to careers in clinical laboratories.

Although the contributory factors giving rise to socioeconomic disadvantage and ethnic minoritisation are complex and multifactorial, they are often outside the control of the student and certainly do not define their ability or promise. However, as scientists and educators, we can proactively mediate measures to mitigate some of the disadvantage to help broaden participation in our professions. Some of the barriers that those from lower socioeconomic backgrounds can be levelled through progressive government policies, including early intervention, aimed at improving quality of education at late primary school [24], grants and scholarships [25], and staff led interventions including mentoring programmes and other academic and social supports [26]. Beyond national policies and government strategies, there are activities that professional bodies representing clinical laboratory professionals and universities can take to incentivise these careers and act as motivating factors for students to continue their studies.

Professional bodies can have outreach activities to increase visibility of clinical laboratory professions on offer and offer advice on career pathways and progression. The UK Institute of Biomedical Science (IBMS) runs a long-standing outreach programme called “Harvey’s Lab Tours” where children and teenagers get the opportunity to participate in the clinical laboratory and understand more about where their samples go and how they are analysed [27]. Although the scope and nature of this programme is primarily focussed on patients and helping them understand the processes in pathology laboratories, the outreach, educational value, and increasing awareness of these professions can be used as a blueprint of how these programmes can be expanded to other young people including from socioeconomically disadvantaged backgrounds. Universities must also take an active role in promoting degree programmes and developing programmes that increase uptake from a broad spectrum of students. There are several approaches that have proven to be effective in bridging the gap to university, beyond schoolwide outreach programme discussed earlier, other proven activities include targeted summer school syllabus and mentoring programmes. The delivery of summer school scientific and laboratory-based programmes, where students can get a taster curriculum of biomedical science, including laboratory experience, have been shown to substantially increase attendance at a university level programme. This was demonstrated in a 2020 study which had a focus on biomedical and medical sciences demonstrated that their interventions increased the proportion to 87% of high-school level students from disadvantaged areas progressed to a 4-year degree [28]. The role of mentoring schemes in widening participation and access to universities has been extensively researched and has shown that well-constructed and administered mentoring schemes are successful in widening access to universities for students from disadvantaged backgrounds and for those from ethnic minorities [29–31].

Access or foundation routes into biomedical focussed courses can play a very important role in closing the entry gap into the discipline. There are hundreds of Bachelor of Science degrees in Biomedical Sciences (including Biomedical Science, Clinical Sciences and core specialities, e.g., Biochemistry and Microbiology) available to students in the UK alone, with many offering foundation year route of entry, often with a lower entry tariff than the non-foundation option. These routes may offer students the opportunity to close the 19.1-month gap that begins to emerge at GCSE levels and enhance their academic skills and knowledge before entering year 1 of their chosen degree. The value of foundation year for lower socioeconomic cohorts is highlighted in a recent UK study which showed that students from lower Participation of Local Areas (POLAR) classifications had a significantly lower attrition rate if they participated in foundation year than if they continued directly to the three-year degree programme [32]. The Foundation year and other access options are integral to improving participation by minority groups in Biomedical Science education and beyond, it is essential that these programmes are maintained and strengthened to help widen participation by as broad a cohort in Biomedical as possible.

A further option which should widen participation is the pursual of a degree apprenticeship in Biomedical Science. Degree Apprenticeships (England and Wales), Graduate Apprenticeships (Scotland), or higher-level degrees (Northern Ireland) offer students to gain a qualification whilst gaining work experience. These apprenticeships are regulated and standardised qualifications with the learner gaining a level 6 (Bachelors) or 7 (Masters) qualification on successful completion of the course and graduate without a student loan/debt. By design, it should be the case that an apprenticeship route should result in significant widening of participation, as the candidate can earn while they learn and are entitled to protected time of at least 0.2 whole time equivalence to support their learning [33]. This route to a degree is relatively new, having been rolled out in 2016, however there have been a series of graduation cycles at this stage so we can begin to assess their impacts on widening participation. The numbers of learners enrolling in degree apprenticeships have increased year-on-year, with 46,800 new starters for 2022/23 a near fold-fold increase compared to the figures of 2017/18 [34]. The most recent statistics from the English Department of Education (for 2023/24) show that there were 370 new starters in healthcare science practitioner with an integrated degree and a further 290 undertaking qualifications as healthcare science assistants and associates [35]. The figures don’t indicate how many of these apprentices are based in the clinical laboratory, however the English Office for Students (OfS) had a recent call for degree apprenticeships for higher education providers in September 2023, there were 11 Universities who were awarded funding to support Biomedical Scientist apprenticeships across three different calls, called “waves” [36]. This is indicative of growing support and quantity of apprenticeship degrees in biomedical science, but the funding allocated remains small, posing a risk to viability of these programmes. However, whether these apprenticeships actually widen participation is contentious at best, paradoxical at worst. The figures for 2024 indicate that degree apprenticeships attract a higher proportion of older, white, male students who are less likely to be from deprived areas [34]. The figures for healthcare science practitioners, which includes biomedical science cohorts, are more diverse in some aspects, namely, a higher proportion of females who are above 25, however over 90% where of white ethnicity [35]. A recent study found higher diversity among those who were recruited into degree apprenticeships from an existing internal post were more diverse than those who were recruited directly, they were generally older, and many were the first in their family to attain a degree [37]. Degree apprenticeships should offer the opportunity to increase diversity and participation among clinical laboratory scientists but do not appear to currently achieve this, the 11 Universities who have been awarded places for Biomedical Science apprenticeships should begin to improve this. It is evident that more work is needed to increase the awareness of these pathways to more diverse student cohorts and to increase the number of university places in these course pathways.

## Understanding and Overcoming Undergraduate Under-Attainment

Once students begin their undergraduate studies in Biomedical Science, they should encounter an intellectually challenging programme and need to gain significant broad-based knowledge relating to professional competencies, pathology specialities, and technical skills. If students are not appropriately supported on this journey, they may not thrive academically demonstrated by graduating with a low honour (i.e. 2.2), or non-honours degree award or leave the programme without the desired terminal award. Black, Asian and other minority ethnic students, especially Black students, are more likely to not complete their degree and are also less likely to attain an upper grade (i.e. 2.1- or first-class honours awarded degree) [38, 39]. This is a long-standing issue, with a report from over 20 years ago indicating that only 2.9% of Black Caribbean students, 3.3% of Black African students, 6.6% of Indian attained a first-class honours degree compared to over 8.9% of Chinese and 10.7% of white students [40]. The most recent figures (2021/2022) for the UK saw a reversal of the closing gap trend, with the first increase in the awarding gap between since 2014/15. There was a 10.7% ethnicity awarding gap [41]. However, the overall trend of improvement among ethnicities obscures a complete failure to address the gap for some ethnicities, particularly for Black Caribbean compared to white students, where the first class or upper second-class honours gap was 24.7% in 2004 and in 2021/22 was only marginally lower at22.6% [41]. There is a need for targeted improvements in this area to significantly improve this long-standing attainment gap. The figures for Biological and Sports Science (BSS) as a subject set show the attainment gap between White and other ethnicities student cohorts to be lower than the total undergraduate population at 8.7% [41], however the attainment gap for BSS is not broken down by ethnicities so the subject set figures may mask a higher attainment gap between certain ethnicities. A London study examined potential contributory factors to explain the attainment gap for biomedical science and medical students and found that there were several contributory factors including social factors such as accent and cultural background potentially contributing to a perceived sense of being different from their peers impacting social cohesion among the greater student body [42]. There are several barriers that have resulted in the attainment gap, including social factors raised in Claridge’s study, other hygiene factors including psychological needs not being met as a result of dissatisfaction with course content, caring responsibilities and needing to engage in part-time employment all contributed to nearly all ethnically minoritised students feeling that their psychological needs weren’t being met in a recent study [43].

A key factor in reducing the attainment gap and thus improve students’ postgraduate opportunities for students of ethnicities other than white, is to identify why these students are more likely to not be engaged with the biomedical science curricula. A new study aimed to shed further light on the key barriers facing ethnic minority healthcare students in Hong Kong, with a large subset of medical sciences (31.25%). They found that obstacles included language barriers, exclusionary social factors, and from religious practices [44]. These obstacles have been recognised and acknowledged for decades but still appear to be among the biggest barriers to bridging the attainment gap [45]. One approach to address which may level many of these barriers is through decolonising or diversifying the curriculum, make a biomedical science curriculum that is tailored for the audience. Through decolonising and diversifying the curriculum, we acknowledge the fact that much of the learnings in medicine and medical science have come about because of exploitation and bias towards maintaining control and prestige by western countries and institutions [46, 47]. It appears that there needs to be a shift from “white people” being the default in the study of health and disease towards a more inclusive and representative curriculum.

One approach to diversify a biomedical science programme was described by a cross institutional west of England research group, the “three R” approach. The “R’s” are Rediscovery; essentially critically analysing the sources of “common knowledge”, intergenerational knowledge isn’t necessarily accurate and unlikely to be representative, Representation; meaningfully embedding and threading diversity throughout the curriculum, and finally, Readiness; being led by students and their personal perspectives and experiences, harness their energy and enthusiasm for change to be the driver to deliver diversification across the curriculum, including sufficient institutional and staff support [48]. The Quality Assurance Agency for Higher Education in the UK (QAA) is an independent body that oversees the quality and standards of higher education institutes including the publication of discipline specific guidance. In 2023, the QAA updated their guidance on Biomedical Sciences and Biomedical Sciences to include specific requirement for the inclusion of Equality, Diversity and Inclusion to be embedded throughout the curriculum of biomedical science(s) degree programmes. The updated standard includes practical tips for implementation of same, including recognition of scientists that are diverse, critical engagement with the history of science and scientific discovery and the creation of an environment that minimises risk of social exclusion based on some of the factors discussed above [49]. These are some of the approaches that have shown to be effective in closing the attainment gap. There are other approaches that appear to be successful, but “*the most important step towards diversification and decolonization is the first one*” [50].

There has been a massive increase in the use of Artificial Intelligence (AI) and generative Artificial Intelligence (genAI) throughout the spectrum of education in recent years [51]. There are well described benefits of AI in education such as facilitating personalised education [52], breaking down language barriers [53], utilising genAI to breakdown complex topics into a language that is familiar to the student and through the development of immersive scenarios for healthcare students [54]. These positives offer a snapshot of some of the ways that AI and genAI can help enhance and improve education for underrepresented student cohorts. However, there are aspects of AI and genAI that can be disadvantageous to students from lower socioeconomic backgrounds. The primary risk that comes with increased use, power, and diversity of genAI models in higher education is equity of access [55]. The appropriate use of genAI to support learning, especially when the educator has been involved has shown that genAI has been shown to be an important tool in improving educational outcomes [56, 57]. Many of the more powerful and complex genAI tools are now subscription services [58], meaning that they are not available to all students equally. Many universities have implemented some sort of AI policy with wide variations in depth and scope [59, 60]. A scale devised by Perkins et al., is a useful guide for academics to provide guidance to students surrounding the level of assistance from AI that they can use that is permissible for assessments, to promote responsible and meaningful engagement with genAI, whilst discouraging inappropriate uses, for example, where genAI develops whole bodies of work on behalf of the student with little or no student involvement [61]. Without these interventions and policies acknowledging the influence that AI and genAI will have on education into the future, the quality of education, learning and acquisition of skills by students may suffer greatly. It is essential that educators give their students the means and skills to engage with AI in a meaningful way, otherwise it can be misused which will result in cognitive costs [62]. This is especially important in the context of biomedical science, where a student must develop core knowledge and skills to enable practice that does not pose risks to patients. Those that can afford to, will benefit from having appropriate access to more powerful tools may have enhance learning of topics which will impact final grades and degree awards. If it is not used appropriately and free at point of use, genAI may further exacerbate a long-standing attainment gap for minoritised and socially disadvantaged students.

## The Road to Registration

A student after completing a Biomedical Science or Sciences degree has a plethora of career options to choose from, including working as a Biomedical Scientist or other healthcare scientist in healthcare and hospital laboratories, industry, veterinary laboratories, research, and further education, to name a few. The work of scientists who practice within healthcare providing laboratories are often regulated and protected titles. Across Europe, 25 nations have regulations in place for the generic profession of “Medical/Biomedical laboratory technician”, in the UK these are Biomedical or Clinical Scientists and these are regulated by the Health and Care Professions Council (HCPC) [63], in Ireland they are Medical Scientists and are regulated by CORU [64], and across many European nations the title most commonly include “technologist” or “technician” with Medical or Biomedical prefixes/suffixes [65].

The European Commission Regulated Professions Database indicates that all bar one country (Lichtenstein) requires a minimum entry requirement of a post-secondary education of 3–4 years (categorised as a PS3- Diplomas post-secondary level), indicating a high minimum level of education required across the continent [65]. Furthermore, most nations mandate a traineeship [11], a further 9 state it is not mandatory. Although, at least two of these nations have mandatory training, either gained during tertiary education (Portugal, where two semesters are spent in a clinical laboratory undergoing training) or the UK, where it is compulsory to have completed core training prescribed by the Institute of Biomedical Science (IBMS) to apply for HCPC registration as a Biomedical Scientist [66]. For the remaining countries, it is not clear whether it is mandated or not. There is a significant body of evidence to support the positive role of training programmes in ensuring that scientists achieve and maintain competence in their respective discipline [67] whilst improving knowledge, job satisfaction, and retention rates [68, 69].

There appears to be a minority of biomedical science graduates that go on to become Biomedical or Clinical Scientists in the UK, with approximately 13% of graduates being employed as Biomedical Scientists 15 months after graduation based on UK HESA graduate attributes data from 2021/22 [70]. As aforementioned, to practice as a Biomedical Scientist in the UK, it is necessary to have both an IBMS approved degree in Biomedical Science and complete a supervised clinical placement in an IBMS approved training laboratory to attain the IBMS Certificate of Competence [66]. This ensures the trainee Biomedical Scientist successfully acquires all learning outcomes prescribed by the IBMS training portfolio to ensure adherence to the HCPC standards of proficiency [63, 71]. According to the IBMS website, there are 64 IBMS accredited undergraduate course providers across the UK, with forty-three of these offering either a sandwich and/or placement component [72]. The number of places available to students who wish to complete a clinical placement during their undergraduate degree in the UK do not appear to be publicly accessible through UCAS nor the IBMS. A recently published report by the HCPC reported that there were 5,058 newly registered Biomedical Scientists from the UK from Sept 2013-Aug 2019 and 2,016 Clinical Scientist from the UK from Jul 2013 to Jun 2019 with greater than 90% of Biomedical and Clinical Scientists maintaining registration 36 months after initial registration [73]. These retention figures suggest that clinical laboratory scientists are successfully finding work and are motivated to stay in post once they get there and have higher retention rate compared to other professions regulated by the HCPC including physiotherapists, speech and language therapists, dieticians, chiropodists/podiatrists and four other regulations professions [74]. The NHS indicates that there are more than 40,000 posts in biomedical science in England [75]. At the time of writing, a search of “jobs.nhs.uk”, “nhs.wales/nhs-jobs-in-wales/”, “jobs.scot.nhs.uk” and “jobs.hscni.net”, using search terms “Biomedical Scientist” and “Scientist” revealed a total of 81 open vacancies for roles clinical laboratories (note, other scientific roles have not been counted, e.g., nuclear physics, physiologists, etc). These are summarised in Table 1 by open vacancies in clinical laboratories according to grade and location of job posting. There are more open positions advertised via recruitment websites, however due to duplication of posts across different sites/providers, it is difficult to assess the true number of vacancies. Nonetheless, and to add context, at the time of writing one major recruitment agency had 92 Biomedical Scientist positions advertised at the time of writing, indicating that there are likely as many locum positions available at any given time as there are longer term openings. These findings demonstrate an on-going shortage of Biomedical Scientists and to a lesser extent, Clinical Scientists in the current system.

**TABLE 1:** Biomedical and clinical scientist advertised vacancies in the NHS as of 10/10/2025.

NHS Regions	Band 5	Band 6	Band 7	Band 8 (a-d)
NHS (England)	13	31	13	11
NHS (Wales)	0	1	1	0
NHS (Scotland)	1	3	2	0
NHS (health and social care jobs in Northern Ireland)	3^a^	1	1	0

^a^ Two were advertised as either band 5 or 6.

The UK, unlike academic routes in Portugal or Ireland, does not guarantee placements for Biomedical Science students that wish to complete them, nor, if a student secures an appropriate placement, is there a certainty of payment to the student for completing their training. These issues are considerable barriers to success for many students, favouring those from higher socioeconomic backgrounds [76]. A recent scoping review highlighted the impacts that unpaid mandatory placements have on healthcare students, including that attendance at unpaid placement resulted a dual financial burden due to costs associated with getting to the placement coupled with the loss of income that may have been generated by the student had they not had to attend the placement on a full time basis, these monetary pressures were significant contributors to mental health threats including anxiety, depression and burn-out [77]. Where most countries are experiencing rising costs of living, unpaid placements will impact the attractiveness and viability of pursuing a profession that mandates this type of training [78] this may lead to a reduction in the numbers of much needed scientists of the future. At the time of writing, there were no training posts for Biomedical Scientists advertised on NHS websites, accentuating what is arguably the biggest barrier for graduates that wish to progress to a career as a registered Biomedical or Clinical Scientist. The lack of paid training opportunities disproportionately affects those from lower socioeconomic backgrounds and underrepresented ethnic minorities [79], which may, unfortunately, close the door on a profession as a Biomedical or Clinical Scientist. This should be considered a disservice considering the barriers that these prospective scientists have already overcome to complete their degrees. This needs to be addressed through national policy and government support to bring the UK in line with other jurisdictions and to broaden participation, whilst also safeguarding pathology services through provision of sufficient scientists to meet demand, thus ensuring service continuity in pathology laboratories in the future.

## Post-Registration, Promotion and Progression

**FIGURE 1:**
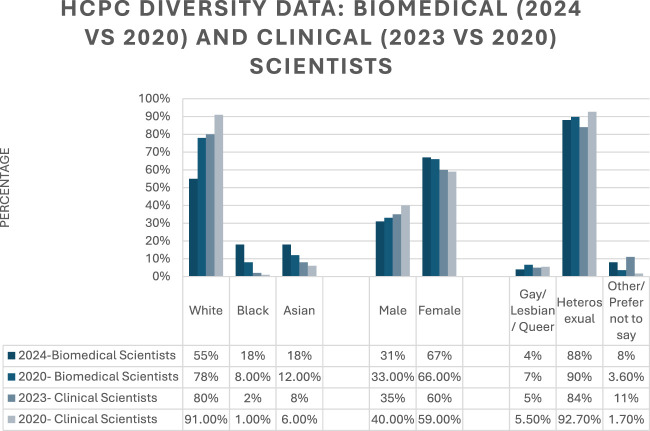
HCPC Diversity Data comparing key ethnic, sex and sexual orientation among Biomedical (2020 vs. 2024) and Clinical (2020 vs. 2023) Scientists.

The HCPC gathers and disseminates findings under the nine protected characteristics under the Equality Act, 2010 (UK), the most recent findings for Biomedical Scientists [4] and Clinical Scientists [3] indicate increased diversity in nearly all metrics when compared to figures from 2020 [2]. Figure 1 summarises key metrics for ethnicity and genders for the two titles. There appears to have been a marked increase in registrants of ethnicities other than white between 2020 and the most recent reports, particularly among Clinical Scientists, where previously over 90% of registrants were white. However, there appears to be room for improvement with regards to sexual orientation, where the figures remain stable between the two timepoints, with c.90% of registrants identifying as heterosexual. The 4%–5% of registrants that identify as lesbian/gay/bisexual compares favourably with the general population of approximately 3.3% as of 2022 [80]. Furthermore, there was a 42% reduction in Biomedical Scientists that identified as Lesbian/Gay/Bisexual between 2022 and 2024 [4]. These figures appear to be buffered within the increased “prefer not to say” or “other” categories, which may suggest that registrants are uncomfortable indicating their sexuality to their regulatory body. Further work needs to be done to ensure that sexual minorities do not feel ashamed or feel they must hide their sexuality at the risk of being exposed to workplace discrimination including microaggressions or insults. This is an especially important consideration in the wider healthcare workforce context where several studies have demonstrated that there is consistent stigma and discrimination among healthcare professionals towards LGBTQ+ patients which may lead to poor patient outcomes [81, 82]. There is a static 2:1, female to male ratio for Biomedical Scientist registrants and a moderately smaller 60:40, female to male ratio for Clinical Scientists. The sex spread is an important factor when considering opportunities for promotion for females and males, which will be examined later in this article.

The benefits of a diverse workforce in a healthcare environment include better decision making, inform culturally appropriate care and expands health research [9, 10]. Thus, the improvement in diversity among ethnicities is welcome, despite the challenges discussed earlier. However, it is important that this trend continues, the work is not complete yet as there are obstacles that still haven’t been overcome, and other challenges are emerging. The HCPC figures represent the total number of registrants and not a breakdown by grade, i.e., whether more junior positions are disproportionately occupied by employees of ethnicities other than white, when compared to promotional level opportunities. These is a growing body of data from the US that although the diversity gap appears to be closing among healthcare staff but that another is opening, one between senior positions and entry level, whereby many non-Caucasian workers occupy positions that are considered entry level and face serious difficulties in attempting to progress their career [83, 84].

### Access to post-graduate education as a barrier to post-registration promotion?

A postgraduate qualification at Masters or Doctoral “D-level”, or equivalent, is most often a necessity for many promotional vacancies in careers across biomedical sciences. This is highlighted by all twenty-four posts at Band 7 or 8 summarised in Table 1, requiring at least a master’s level qualification or equivalent. This may be due to a lack of awareness of postgraduate research options or other access barriers among ethnic minorities. A 2023 study showed that up to a third of minority ethnic final year students not being aware of available research pathways and 40% of those from Arabic or Middle Eastern backgrounds not being aware of postgraduate research study pathways [85]. An examination of the barriers to pursuing postgraduate research reveals that over 60% of ethnic minorities feel that they are likely to be unsuccessful in applying to research intensive universities and nearly half of ethnic respondents cite the lack of role model PhD supervisors as a barrier to pursuing research [85]. These factors are likely related, highlighted by lower representation of minorities among academic staff coupled with inherent biases in the curriculum in favour of white scientists, academics, and students [86, 87]. It has been known for at least a quarter of a century that ethnic minorities are underrepresented among university faculties, where it was then estimated that non-white academics accounted for 5.5% of staff [88]. There have been inroads in the intervening years, where 22% of academic posts are occupied by non-white ethnicities (where ethnicity has been reported) [89]. However, there remains significant room for improvement when examining senior academic positions, especially for black academics, occupying 1% of Professorial or other senior academic roles [89]. There are several benefits to having more diversity in academic staff, including more effective communication with minoritised students [90], a deeper understanding and subsequent adaptation of teaching to account for inherent barriers experienced by ethnic minorities [91] and acting as role models [92]. The lack of ethnic representation among higher positions in academia results in lower confidence in students in their belief that they can achieve this too as there is no pathway to follow [92]. The inclusion of more ethnic diversity has recently been shown to improve student outcomes academically and subsequently in the workforce, importantly this study showed that the inclusion of more diversity improved student outcomes for minoritised student and did not negatively impact the outcomes of white students [93]. These factors are most relevant to students wishing to progress to research shortly after undergraduate graduation. However, there is a large proportion of Biomedical Scientists who pursue postgraduate qualifications after gaining experience in a core discipline or speciality, for these learners, other challenges exist.

Clinical laboratory scientists who have gained experience and specialisation may wish to seek a higher degree to complement their work experience, many of whom may have been out of third level education for a significant period and face distinct challenges as well as those already discussed. These learners or researchers are most often working as well as pursuing a postgraduate qualification which has been shown to lead to sacrifices (including in one’s personal life), increased anxiety and stress and higher risk of burn-out than the greater working population [94, 95]. The balance between home responsibilities and post-graduate studies have shown to be major contributory factors in holding students back [96]. Most of the clinical laboratory scientific workforce are women and it is a well-established fact that the majority of caring activities still fall with women, despite advances in paternal and parental leave, and the unfair, negative impact that these can have on women’s progression opportunities and salary [97–99]. Working mothers who wish to pursue a doctorate level qualification face particularly unique challenges, including feeling isolated without support and impacting on their physical and mental wellbeing [100, 101]. This is something that needs to be addressed urgently, if we are to see a widening of participation in senior roles by women, particularly when they are predominant in the workforce. There are practical steps that can be undertaken to remove or at least lower some of these barriers by institutes (both healthcare and educational). These include facilitating a personalised approach to personal and professional development, policy development that explicitly acknowledges and provides supports for women, particularly mothers who wish to undertake research while working and finally the development of diverse support networks and forums for researchers to develop connections and ameliorate that sense of loneliness [101]. Experienced clinical laboratory scientists who also have life experience, especially mothers, can add huge value to the research and knowledge ecosystem and need to be fully supported in work and academia to reach their fullest potential.

The HCPC gathers information for all registrants but does not break this down into different grades or categories depending on job title, responsibilities, or salary bands/grades. Therefore, there is a dearth of data relating to the makeup of promotional grades regarding gender, age and ethnicity in the UK and Europe. A comprehensive clinical laboratory workforce study from the US showed that White workers clinical laboratory workers occupy an increased proportion of roles as you move up through grades occupying 55.3% and 54.7% of roles at Medical Laboratory Technician (MLT) and Scientist (MLS) roles levels respectively (salary averages in 2017 for MLT were $45,715 and $61,112 for MLS) whilst occupying 41% of Medical Laboratory Assistant roles attracting an average salary of $37,772 [102]. This is a picture that appears to be echoed in the UK and Europe in healthcare professions more broadly. A study published in 2025 of the NHS workforce consisting of over 5,700 participants, including 480 Healthcare scientists, showed that Asian and Black healthcare workers had less chance of occupying a higher salary grade than their white counterparts following adjustment for sex, education, and occupation. The figure was most marked for Black workers from oversees compared to white UK born colleagues, where the former were half as likely than the latter to occupy a higher graded post [103]. Consultant Clinical or Biomedical Scientist is the highest grade of clinical laboratory scientist in the UK. This role commonly requires the holder to be qualified to “D” level, most commonly PhD and be a Fellow of the Royal College of Pathologists in their given speciality, usually a two-part curriculum with associated examinations [104]. There are no specific figures for diversity of holders of consultant level scientific roles in the UK, however, there remains a persistent diversity gap among consultant grade physicians in the UK, with 41% of consultants from an ethnic minority background compared to 64.3% of non-consultant doctor positions being occupied by ethnic minorities [105]. There needs to be a focussed study on the composition of leadership positions in laboratories to assess if there is an existing and/or developing gap.

## The Future

**FIGURE 2:**
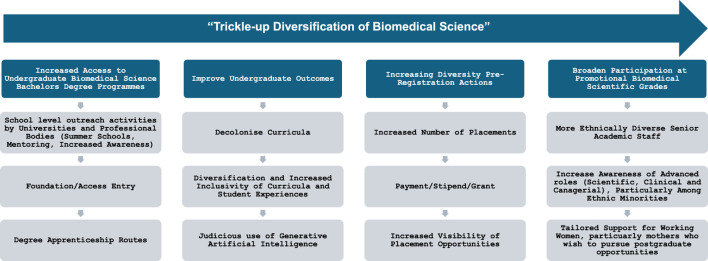
A trickle-up approach to diversity and widen participation in biomedical science.

The clinical laboratory of the future will likely have more instrumentation, computation, and automation; however, the scientist will still be present. The role of the clinical laboratory scientist has shifted considerably since its origins, from a purely technical to technical specialist, advisory and clinical role [8]. AI will likely play an important role in reducing pre-analytical, analytical, and post-analytical errors and automating more processes [106]. The increasing use of AI in the workplace, including the clinical laboratory, can offer the opportunity to enhance productivity with more focus on consequential tasks [107]. A meaningful embrace of AI in the clinical laboratory may actually play a role in increasing the number of scientists, especially those conducting high level and consequence activities, as demonstrated in other industries [108]. The UK and to a lesser extent, the US, have embraced advanced roles of laboratory scientists to “Consultant” level in the UK and “Laboratory Director” in the US, although less than 10% of Laboratory Directory roles are occupied by non-physicians [109]. A more diverse, well-educated clinical scientific workforce is essential to meet future demands of the profession, to respond and implement new technologies, advanced testing algorithms and personalised medicine to ensure patient safety. The demands on healthcare systems are continuing to grow; marked by an ageing population with multiple co-morbidities and increased case complexity [110], The most sustainable and promising way to ensure this is through recognising the current barriers that are present at undergraduate level, pre-registration, and post-registration barriers to progression The key findings of this review have been summarised in Figure 2, demonstrating a sustainable, step wise, “trickle-up” approach to diversifying biomedical sciences at each of the primary hurdles. Although the core foci of this review have been improving participation by ethnic minorities and those from disadvantaged backgrounds, these steps can be tailored and applied to improve diversity among other minoritised cohorts also.

Interventions at secondary level of education are needed to increase awareness of clinical laboratory professions. Universities and professional bodies can play an active role in this through the provision of meaningful outreach programmes, mentoring, and, facilitating early introduction to laboratory activities, especially for students from socioeconomically disadvantaged areas. The provision of increased access routes, especially foundation year, can also increase diversity among undergraduate biomedical science students. Once at undergraduate level, supports and technological advances can level the playing field in terms of attainment and reducing attrition, once these are supported and overseen appropriately. The key barrier to widening participation seems to be gaining professional registration, where a majority of students who have undertaken bespoke, tailored programmes, many of which are accredited, appear to not join the profession. The key challenges that should be dealt with as a priority include the lack of integrated placement years in degree programmes, competition for training places being offered by clinical laboratories and economic challenges associated with undertaking a non-paid placement. There needs to be dedicated training places to a higher number of students in the UK, ideally supported with a stipend or payment. There is a distinct lack of data surrounding diversity regarding the workforce at promotional grades in clinical laboratories, this needs to be addressed to assess if there is a gap as there are in other healthcare professions. Regardless, there needs to be further supports for Biomedical and Clinical Scientists wishing to undertake postgraduate learning and research, particularly for mothers and ethnic minorities, to ensure that the workforce is futureproofed and can respond appropriately to increasing clinical demand.
